# Systematic Evaluation of DBS Parameters in the Hemi-Parkinsonian Rat Model

**DOI:** 10.3389/fnins.2020.561008

**Published:** 2020-10-09

**Authors:** Soheil Mottaghi, Oliver Buchholz, Ulrich G. Hofmann

**Affiliations:** ^1^Section for Neuroelectronic Systems, Department of Neurosurgery, Medical Center – University of Freiburg, Freiburg, Germany; ^2^Faculty of Medicine, University of Freiburg, Freiburg, Germany; ^3^Technical Faculty, University of Freiburg, Freiburg, Germany

**Keywords:** systematic DBS evaluation, induced DBS rotation, transient rotation, therapeutic DBS frequency, DBS waveform, DBS amplitude, NES STiM, Unilateral 6-OHDA PD Model

## Abstract

Electrical stimulation of the subthalamic nucleus (STN) is clinically employed to ameliorate several symptoms of manifest Parkinson’s Disease (PD). Stimulation parameters utilized by chronically implanted pulse generators comprise biphasic rectangular short (60–100 μs) pulses with a repetition frequency between 130 and 180 Hz. A better insight into the effect of electrical stimulation parameters could potentially reveal new possibilities for the improvement of deep brain stimulation (DBS) as a treatment. To this end, we employed single-sided 6-hydroxidopamine (6-OHDA) lesioning of the medial forebrain bundle (MFB) in rats to systematically investigate alternative stimulation parameters. These hemi-parkinsonian (hemi-PD) rats underwent individualized, ipsilateral electrical stimulation to the STN of the lesioned hemisphere, while the transiently induced contralateral rotational behavior was quantified to assess the effect of DBS parameter variations. The number of induced rotations during 30 s of stimulation was strongly correlated with the amplitude of the stimulation pulses. Despite a general linear relation between DBS frequency and rotational characteristics, a plateau effect was observed in the rotation count throughout the clinically used frequency range. Alternative waveforms to the conventional biphasic rectangular (*Rect*) pulse shapes [Triangular (*Tri*), Sinusoidal (*Sine*), and Sawtooth (*Lin.Dec.*)] required higher charges per phase to display similar behavior in rats as compared to the conventional pulse shape. The Euclidean Distance (ED) was used to quantify similarities between different angular trajectories. Overall, our study confirmed that the effect of different amplitude and frequency parameters of STN-DBS in the hemi-PD rat model was similar to those in human PD patients. This shows that induced contralateral rotation is a valuable readout in testing stimulation parameters. Our study supports the call for more pre-clinical studies using this measurement to assess the effect of other DBS parameters such as pulse-width and interphase intervals.

## Introduction

Deep brain stimulation (DBS) has matured over the last decades toward a valuable interventional tool to treat a number of neurological and even psychiatric illnesses: Parkinson’s disease (PD) and dystonia have been true ice-breaker applications (Benabid et al., 1991; Tronnier et al., 2002, 2015; Vidailhet et al., 2005; Hardesty and Sackeim, 2007; de Hemptinne et al., 2015), alleviation of chronic pain (Russo and Sheth, 2015) and seizure reduction in epileptics (Velasco et al., 2000; Vonck et al., 2002) were added subsequently. Most recently, obsessive compulsive disorder (OCD) (Alonso et al., 2015), Gilles-de-la-Tourette’s syndrome (Almeida et al., 2015) and major depression (Schlaepfer et al., 2013) have been established as targets as well. In all cases, electrical stimulation is applied to a brain region specific to the disease by implanting noble metal electrodes. Electrical parameters are then empirically adjusted to obtain the best treatment outcome. A range of stimulation frequencies (*f*), amplitudes (*Amp.*), pulse-widths (*PW*) and pulse shapes have been investigated over the years (Moro et al., 2002; Spieles-Engemann et al., 2010; Gimsa et al., 2011; So et al., 2012; Kang and Lowery, 2014; Khoo et al., 2014; Vallabhajosula et al., 2015; Picillo et al., 2016; Su et al., 2018). However, testing alternative DBS parameters in PD patients can be challenging for ethical, certification and technical reasons, demanding a more versatile platform to study the biological outcomes of DBS parameters.

The unilateral 6-hydroxydopamine (6-OHDA) rat model is one of the more commonly used PD models and has been selected for this study. Unilateral injection of 6-OHDA, a highly specific neurotoxin, into the medial forebrain bundle (MFB) of a rat causes substantial dopamine degeneration at the injection site (ipsilaterally) (Ungerstedt, 1968). The main feature of this model is that it keeps one hemisphere intact while the other hemisphere loses its dopaminergic connections after the 6-OHDA injection. This model is thought to provide sufficient behavioral and electrophysiological similarities to human PD to make it an appropriate platform for pre-clinical PD studies including DBS. A wealth of studies on DBS to the subthalamic nucleus (STN-DBS) have been conducted over the years on the hemi-PD rat model and have proven its versatility (McConnell et al., 2012; So et al., 2012, 2017; Summerson et al., 2014). In this study, we quantified transient contralateral DBS-induced rotations while varying DBS electrical parameters (amplitude, frequency, and particularly waveform). This electrically induced rotation measure was first described by So et al. (2017) and we realized its potential to yield a reliable and quantifiable criterion to further study the electrical parameter space of DBS.

## Materials and Methods

Female Sprague-Dawley rats (290–330 g; *n* = 9) acquired from Charles River Laboratories were housed for 2 weeks at the animal facility of the University Medical Center in Freiburg prior to any further action. The rats were housed under a 12 h/12 h light-dark cycle and were provided with food and water *ad libitum*. All procedures involving animals were approved by the Animal Care Committee of the University of Freiburg under proposal G15/031 and performed accordingly.

### Electrode Assembly

Bipolar Teflon-coated Platinum/Iridium stimulation electrodes (90% Platinum, 10% Iridium, 50 μm diameter, Science Products GmbH, Germany) were prepared in-house. A 10 cm wire was folded in half and twisted 13 times with a tetrode maker (Tetrode Twister, LabMaker, Germany). Two pins were soldered to the wires and the impedance of the electrodes was checked in a saline solution (0.9% NaCl, Sigma Aldrich GmbH, Germany) in order to ensure the usability of the electrodes prior to implantation. All implanted electrodes had an impedance <5 kΩ. The electrodes were disinfected using 70% ethanol and kept in a clean container until the surgery.

**FIGURE 1:**
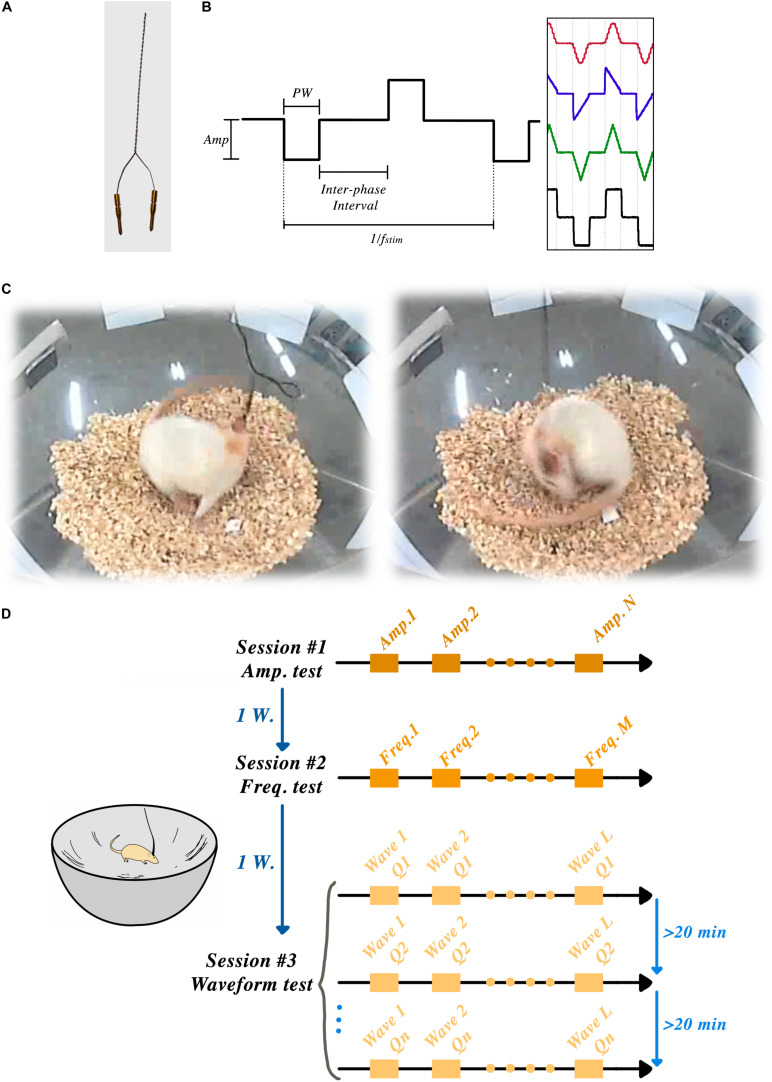
STN-DBSparameter tests: **(A)** Custom-made bipolar stimulation electrodes were implanted into the STN region, ipsilateral to the lesioned side. **(B)** Amplitude, frequency, pulse-width, interphase interval and wave-shape were the parameters that could be set in each session. **(C)** Two frames of a rat showing induced rotation during the STN-DBS. **(D)** Schematic timeline of the experiment showing three sessions and one week of rest between each session.

Figure 1A shows an example of a bipolar electrode.

### Handling and Surgery

Prior to surgery, all rats underwent several days of handling in order to familiarize them with the experimenter and the test apparatus. Rats were anesthetized with oxygen (0.15 l/min) and isoflurane (Abbvie, United States) through an appropriate nose cone; the concentration of anesthetic was initially set to 4% and gradually lowered to 1.5% after placing the animal into the stereotaxic frame (David Kopf, United States). Animal breathing, reflexes and level of anesthesia were monitored throughout the duration of the surgery. Aseptic surgery techniques were applied as described in Richter et al. (2013) and its supplemental video.

Under anesthesia, animals were either lesioned by an injection of 6-OHDA or implanted with the pre-fabricated electrode assemblies at the coordinates shown in the following tables (Tables 1, 2). For these procedures, a 0.8 mm ø hole was carefully drilled with a handheld dental drill at the intended implantation site, through which the dura was resected using a fine needle. The micro-injector or electrode assembly, mounted to the frame’s micromanipulator, was subsequently lowered manually into position at a rate of approximately 200 μm/s, and the skull aperture around the implanted electrode was filled with bone wax. Once in place, the electrode was fixed to a nearby stainless-steel screw anchor (0–80 × 1/8; Plastics One) using a 2-compound dental cement (Palapress; Heraeus Holding GmbH; Germany). An upward-facing connector, mounted on a custom-made Printed Circuit Board (PCB), was attached to the electrodes and fixed with dental cement. Sprague-Dawley rats received an analgesic immediately before surgery, and for the four subsequent days (Carprieve, 1 ml/kg s.c.; Norbrook, United Kingdom).

**TABLE 1:** Lesioning coordinates in MFB, quantity of 6-OHDA per injection and injection rate.

	**AP^1^ (mm)**	**ML^2^ (mm)**	**DV^3^ (mm)**	**Quantity**	**Injection Speed**
*MFB*	−4.4 −4.0	−1.2 −0.8	−7.8 −7.2	2.5 μl 3.0 μl	1 μl/min 1 μl/min

*Coordinates according to (Paxinos et al., 1985). ^1^Anterior-Posterior (AP), ^2^Medial-Lateral (ML), and ^3^Dorsal-Ventral (DV).*

**TABLE 2:** Electrode implantation coordinates in M1 and STN.

	**AP (mm)**	**ML (mm)**	**DV (mm)**
*M1*	+2.5	±3.0	−1.6
*STN*	−3.8	±2.4	8.0

### Lesioning

6-Hydroxidopamine solution (3.6 mg 6-OHDA in 1.5 ml of 0.2% ascorbic acid solution in 0.9% NaCl, Sigma-Aldrich GmbH, Germany) was freshly prepared prior to each operation, and kept in ice and in the dark throughout each surgery. All the animals (*n* = 9) received lesioning in the MFB of the right hemisphere (see Table 1). The 6-OHDA solution was injected using a Hamilton syringe (Hamilton, United States) and a microinjection pump (WPI Instrument, United States) under stereotaxic coordinate control. The canula was left in place for 5 min post-injection, allowing the tissue to absorb the injected volume. The animals received postsurgical care for a week, with diluted food provided to them and their food and water intake strictly monitored.

The lesion’s success was assessed with an apomorphine challenge (Tieu, 2011). Animals showing, on average, at least three anti-clockwise rotations per minute after a subcutaneous injection of 0.05 mg/kg apomorphine (Sigma Chemicals, Germany; diluted in 0.2% ascorbic acid) for 30 min post-injection were considered well-lesioned.

A week later, all the successfully lesioned rats (*n* = 9) underwent another aseptic surgery to implant bipolar recording electrodes (ø 50 μm, 90% Platinum/10% Iridium electrodes, Science Products GmbH, Germany) in the primary motor-cortex (M1) and the STN at the coordinates shown in Table 2. All stimulation and recording electrodes were manually assembled under microscope control (see Figure 1A) and stereotaxically implanted in the ipsilateral STN to the lesioned hemisphere (right), as described above.

The reference and ground electrodes were placed above the cerebellum, with the reference electrode implanted sub-durally and the ground electrode connected to a screw fixed in the skull. Similar postsurgical recovery care was provided as that explained in the lesioning section.

Animals were habituated to a corner-free environment (bowl) more than 7 days prior to the experiments. All the electrical stimulation pulses were produced by an open-source, custom-made electrical stimulator: the NES STiM. Detailed system descriptions, as well as a comparison between the NES STiM and common commercial devices, have been previously published (Mottaghi et al., 2020). In brief, the NES STiM is able to generate four independent biphasic arbitrary current waveforms using four dedicated current source modules. It is designed to be integrated and utilized in pre-clinical and experimental studies, while receiving its stimulation parameters from a computer. The parameters that can be adjusted are shown in Figure 1B.

### Statistical Analysis

Pearson linear correlation analysis was conducted on the measurements from each experiment to evaluate the strength of association between the variable under study (amplitude, frequency, and charge-waveform) and the rotational results. Additionally, a significance *t*-test was performed on the results from the waveform experiment to assess the difference between the *Rect* pulses and other waveforms.

### Histology

Animals were deeply gas anesthetized immediately after the last experiment. A transcardial perfusion with 4% formaldehyde (PFA in phosphate buffer) was conducted to fix and then extract the brain. The extracted brains were placed in PFA for 7 days and then stored in a 30% sucrose solution. Coronal sections (40 μm) were cut along the implanted electrodes’ trajectories (Cryostar NX70, Thermo Fischer scientific, United States) and stored on glass slides at −20°C. Tyrosine hydroxylase (TH) and Nissl staining procedures were performed to assess the dopamine loss and electrode positioning in the brain, respectively.

## Results

### Individualized Current Stimulation Threshold

We used the earlier observation from So et al. (2017) that hemi-PD animals respond to electrical STN stimulation (in absence of any pharmacological stimulus) by rotating along their vertical axis – a behavior demonstrated in Figure 1C and usually only seen after chemical stimuli (e.g., the so-called apomorphine challenge, see above).

After connecting the NES STiM to each animal, a personalized threshold *I*_0_ was found for each rat. *I*_0_ was the smallest current amplitude that induced a transient rotation of less than 180° over 30 s. After this calibration, three rats were excluded from further experiments as they demonstrated an overly sensitive response to stimulation, leading to early dyskinetic side-effects. After finding the *I*_0_ for each individual animal, they were given a 30-min break before the actual experiments started. The angular position of the rats was measured using a video recording of their behavior throughout the experiment using the BORIS software (Friard and Gamba, 2016) and further analyzed in Matlab (Mathwork, United States). Figure 1D illustrates the schematic timeline of the experiments and the corner-free environment that each animal was tested.

### Stimulation Paradigm #1: Effects of Variations in Amplitude

**FIGURE 2:**
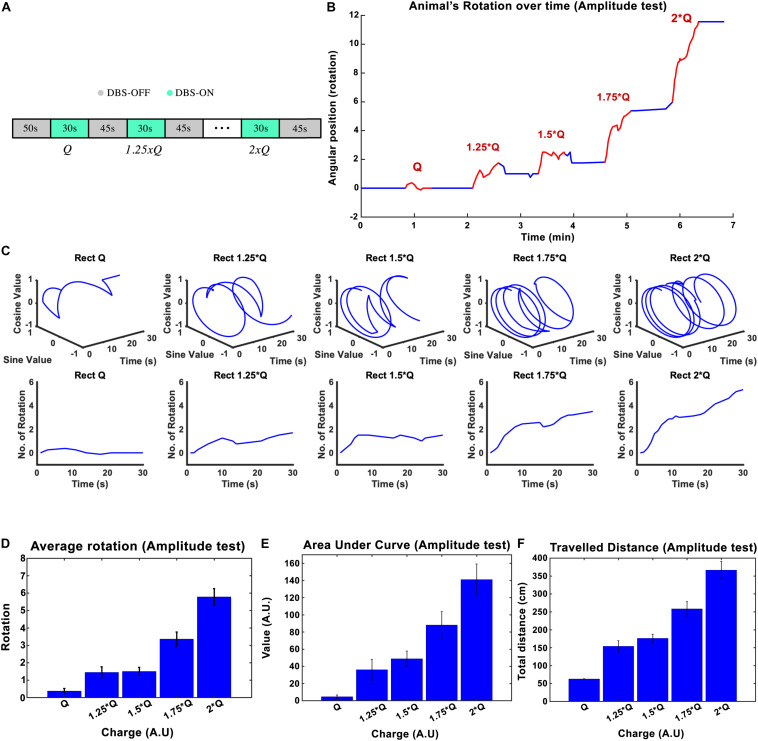
The influence of stimulation amplitude on the induced rotation caused by STN-DBS. Average cumulative angular position of the hemi-PD rats (*n* = 6) with *Q*_0_, 1.25 × *Q*_0_, 1.5 × *Q*_0_, 1.75 × *Q*_0_, and 2 × *Q*_0_. **(A)** The protocol of DBS on/DBS off sequences performed in this experiment. **(B)** Angular position of a particular rat over the course of a complete amplitude test. **(C)** Circular and linear rotational trajectories of a rat over time for each amplitude. **(D)** Average maximum rotation across rats for each amplitude. **(E)** Average area under the rotation vs. time curve for each amplitude. **(F)** The average total distance travelled over the DBS on episodes for each amplitude.

The stimulation amplitude was the first parameter that we tested in this study. Biphasic *Rect* pulse trains with a frequency of 130 Hz, 65 μs PW, and 100 μs interphase interval were applied (see Figure 1B for a representation of the effect of each parameter). During the experiment, an individual rat’s stimulation always started with amplitude *I*_0_ and was followed by 1.25 × *I*_0_, 1.50 × *I*_0_, 1.75 × *I*_0_, and 2 × *I*_0_, with each stimulation episode lasting for 30 s and leaving a 45-s DBS-off period in between each episode (see Figure 2A).

The charge injected in each episode can be calculated as Q = I × t, where I is the current amplitude and t is the duration of the stimulation. The mean ± standard deviation of the total charge injected from *I*_0_ to 2 × *I*_0_ varied from 1.26 ± 0.38 μC/s to 2.53 ± 0.76 μC/s across the six rats that participated in the experiments. A plot of the angular position of one of the hemi-PD animals over the course of the amplitude test is shown in Figure 2B, with blue and red lines representing DBS off and on respectively. In order to better visualize the experiment, the individual trajectories of the rat for each 30-s amplitude test episode are pictured using circular plots in Figure 2C. *I*_0_ caused, on average, not more than a 1/4 rotation, whereas an amplitude of 2 × *I*_0_ induced close to six full rotations during the 30-s episodes (see Figure 2C). Pearson’s linear correlation coefficient, computed on the total number of rotations per 30-s episode, showed a strong linear relationship between the amplitude and the rotations (rho = 0.945, *p*-Val = 0.015) (see Figure 2D). To include the time-dependent dynamic of the rotations, the area under the time-rotation curve was used as a supplementary measure. It showed an increase from 5 to 141 when the amplitude ramped up from *I*_0_ to 2 × *I*_0_, respectively. Clearly, the linear correlation analysis for the area under the curve demonstrated a strong linear relationship with the amplitude as well (rho = 0.977, *p*-Val = 0.004) (see Figure 2E). Under the same testing conditions, the averaged total distance traveled during DBS saw an increase from 62.8 to 366.7 cm with the increase in amplitude, leading to a correlation coefficient of rho = 0.982, and a *p*-Val = 0.003 (Figure 2F).

### Stimulation Paradigm #2: Effects of Variations in Frequency

**FIGURE 3:**
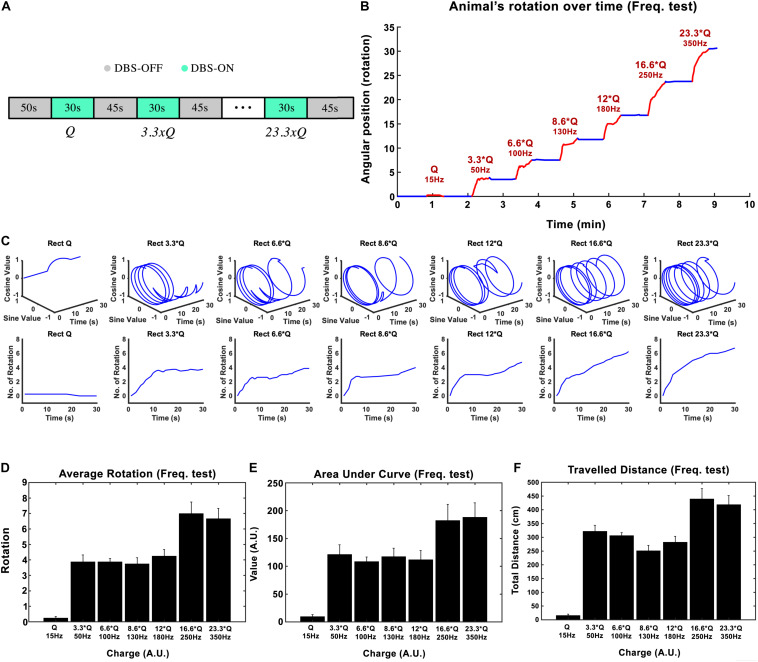
Influence of stimulation frequency on the induced rotation caused by STN-DBS. **(A)** The protocol of DBS on/DBS off sequences performed in this experiment. **(B)** Average accumulative angular position of the hemi-PD rats with 15 Hz (*Q*_0_), 50 Hz (3.3 × *Q*_0_), 100 Hz (6.6 × Q_0_), 130 Hz (8.6 × *Q*_0_), 180 Hz (12 × *Q*_0_), 250 Hz (16.6 × *Q*_0_), and 350 Hz (23.3 × *Q*_0_). **(C)** Circular and linear trajectories of the rotations over time for each charge level. **(D)** Average maximum rotation across rats for each frequency. **(E)** Average area under the rotation vs. time curve for each frequency. **(F)** The average total distance travelled over the DBS on episodes for each frequency.

Subthalamic nucleus-deep brain stimulation frequency was tested in the next experimental session. Biphasic *Rect* waveforms with 65 μs PW and 100 μs interphase interval were applied. The lowest amplitude at 15 Hz frequency that led to an induced rotation of less than 180-degrees over 30 s was defined to be our *I*_0_. The current amplitude was left unchanged at this value for the rest of the experiment. Stimulation episodes with 15, 50, 100, 130, 180, 250, and 350 Hz waveforms were applied, each lasting 30 s, with 45-s pauses in between each episode (see Figure 3A).

In this experiment, the injected charge per second varied from 0.234 ± 0.067 μC/s at 15 Hz to 5.6 ± 3.9 μC/s at 350 Hz across all the rats (*n* = 6). As an example, the angular position of a particular hemi-PD rat over the course of the experiment is shown in Figure 3B. Circular trajectories for each frequency are also depicted in Figure 3C to help comprehend the impact of DBS frequency on rotation. The total rotation resulting from DBS rises from 1/4 rotation at 15 Hz to almost four rotations at 50 Hz.

Let’s consider the injected charge at 15 Hz as *Q*_0_. Although the injected charge increases from 3.3 × *Q*_0_ at 50 Hz to 12 × *Q*_0_ at 180 Hz, the animals’ rotations remain almost comparable (see Figure 3D). This pattern changes when the number of rotations reaches seven at 250 Hz. Increasing the DBS frequency to 350 Hz did not increase the rotations further. A similar pattern was observed when the area under the curve and total distance were calculated (see Figures 3E,F). Linear correlation analysis was performed on all three features (total rotation, area under the curve, and the total distance). The results are presented in Table 3.

**TABLE 3:** Linear correlation analysis results on the three features (total rotation, area under the curve, and the total distance).

**Test**		
Total rotation	rho_*rot*_ = 0.894	*p*-Val_*rot*_ = 0.007
Area under the curve	rho_*AUC*_ = 0.863	*p*-Val_*AUC*_ = 0.012
Total distance	rho_*TD*_ = 0.785	*p*-Val_*TD*_ = 0.036

### Stimulation Paradigm #3: Effects of Variations in Waveform-Amplitude

**FIGURE 4:**
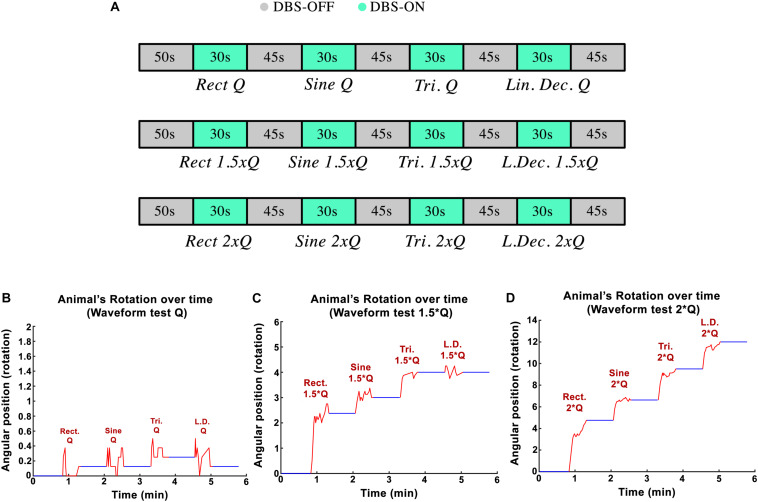
**(A)** Protocol implemented to test variations in waveform-amplitude in each part of the experiment. **(B–D)** Angular position of PD rats during waveform experiments with *Q*_0_, 1.5 × *Q*_0_, and 2 × *Q*_0_ charge intensities.

The goal in the third experimental session was to assess the influence of the stimulation pulses’ shape on the rotational behavior of the hemi-PD rat model. Standard biphasic *Rect* pulses were compared against *Sine*, symmetric *Tri* and Sawtooth (linear decaying, *Lin. Dec.*) waveforms. The *I*_0_ for the *Rect* waveform was titrated in a similar manner as in the amplitude paradigm (130 Hz frequency, 100 μs PW, and 100 μs interphase interval). The equivalent initial current amplitudes, *I*_0_, for the different waveforms were calculated in order to keep the charge *Q*_0_ consistent between waveforms. A sequence of *Rect*-*Sine*-*Tri*-*Lin.Dec.* A total of 30-s stimulation episodes with 45-s DBS-off breaks in between was carried out for *Q*_0_, 1.25 × *Q*_0_, 1.5 × *Q*_0_, 1.75 × *Q*_0_, and 2 × *Q*_0_. A 20-min break was given between charges so that the rats could recover (see Figure 4A). The animals’ average angular position over the course of the stimulation episodes with *Q*_0_, 1.5 × *Q*_0_ and 2 × *Q*_0_ are depicted in Figures 4B–D.

**FIGURE 5:**
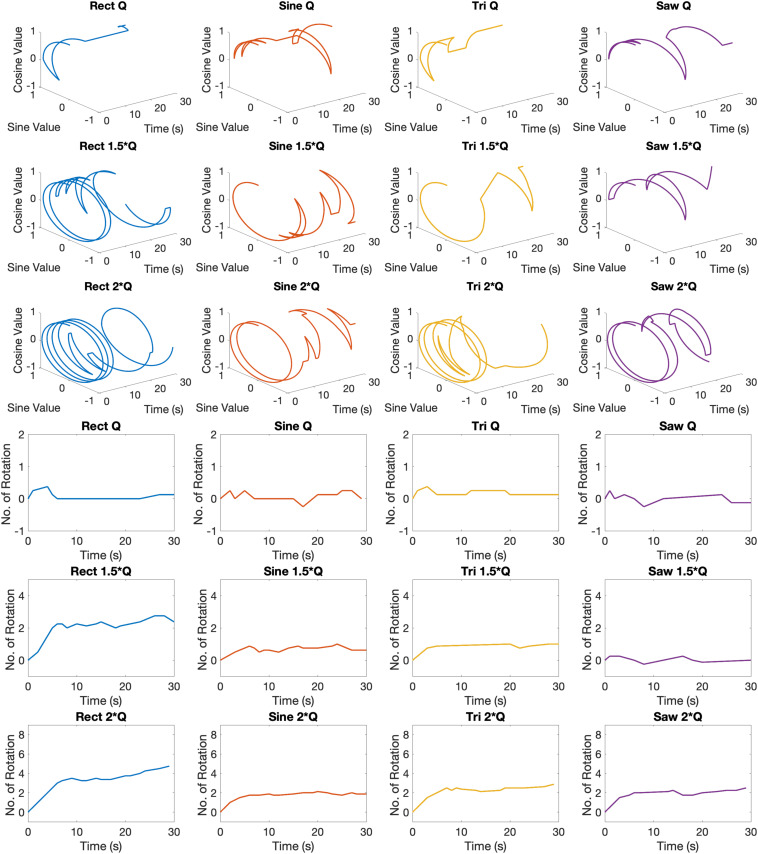
Circular and linear induced rotation trajectories during waveform experiment with *Q*_0_, 1.5 × *Q*_0_, and 2 × *Q*_0_ charge levels.

Examples of circular trajectories for *Q*_0_, 1.5 × *Q*_0_, and 2 × *Q*_0_ charge levels are illustrated in Figure 5. No alternative waveform (i.e., apart from the biphasic *Rect* pulses) could induce a full rotation with a charge level of *Q*_0_ over the course of a 30-s stimulation episode. An additional 50% increase in charge (1.5 × *Q*_0_) resulted in 2.8 rotations for the *Rect* waveform, while yielding only one full rotation for the *Sine* and *Tri* waveforms and only a quarter rotation for *Lin. Dec.* pulses. The 2 × *Q*_0_ charge caused 4.74 rotations on average for *Rect*, 2.12 rotations for *Sine*, 2.87 rotations for *Tri* and 2.49 rotations for *Lin. Dec.* waveforms.

**FIGURE 6:**
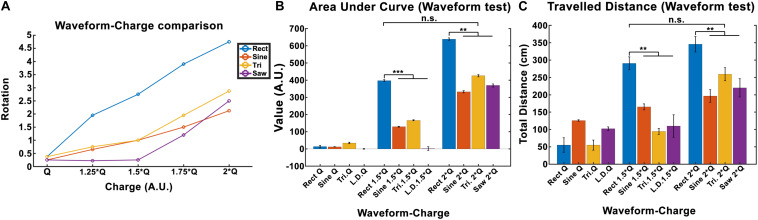
Induced rotation comparison between waveforms (here *Q* is equivalent to *Q*_0_). **(A)** A charge-waveform comparison showed stronger rotation for the *Rect* waveform compared to the *Sine*, *Tri*, and *Lin.Dec*. **(B,C)** Area under the curve and total traveled distance showed significant differences between the standard *Rect* pulse shape and the other pulse-shapes with the same charge. Interestingly, no significant difference was seen between the 1.5 × *Q Rect* pulse shape and the other waveforms with 2 × *Q*.

The total locomotion increases with charge for all waveforms, however, the *Rect* waveform produces a distinctly different response compared to the other three waveforms. As presented in Figure 6A, *Rect* 1.5 × *Q*_0_ and 2 × *Q*_0_ resulted in almost twice as many rotations than the other waveforms, which all showed a comparable performance at these charge levels. The average area under the curve and the total traveled distance are depicted for *Q*_0_, 1.5 × *Q*_0_, and 2 × *Q*_0_ charge levels in Figures 6B,C. Pearson linear correlation coefficients for results from different waveforms are available in Table 4.

**TABLE 4:** Linear correlation analysis results for different waveforms.

**Test**		
*Rect*	rho = 0.993	*p*-Val = 0.00065
*Sine*	rho = 0.9931	*p*-Val = 0.00068
*Tri*	rho = 0.967	*p*-Val = 0.007
*Lin. Dec.*	rho = 0.871	*p*-Val = 0.054

A significance *t*-test performed on the area under the curve and the total distance features showed that 1.5 × *Q*_0_ and 2 × *Q*_0_ *Rect* waveform DBS induced significantly more rotations compared to the other waveforms with the same charge. However, no significant difference between the results of 1.5 × *Q*_0_ *Rect* and 2 × *Q*_0_ of other waveforms was observed (see Figures 6B,C).

## Discussion

A total of 30-s STN-DBS episodes were systematically applied, assessing a variety of stimulation parameters in hemi-PD rats. A better understanding about the effect of each parameter could potentially disclose great opportunities to improve DBS treatments.

**FIGURE 7:**
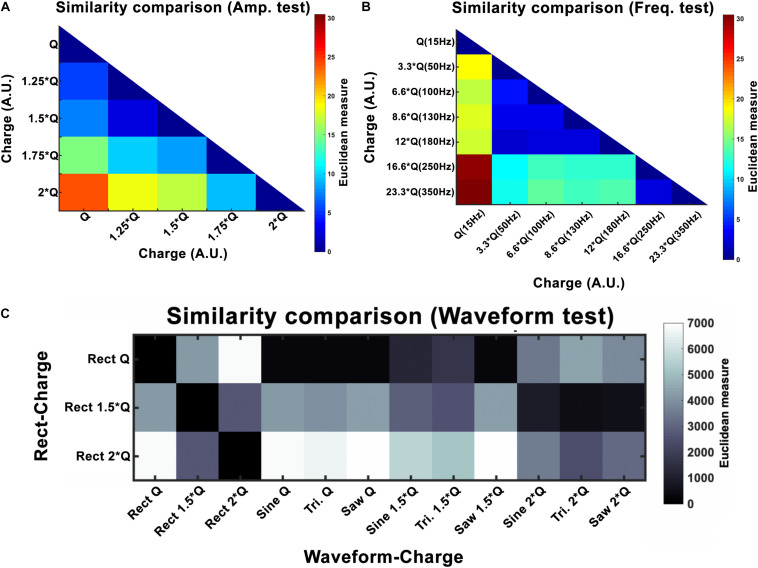
Euclidean distance (ED) for amplitude **(A)**, frequency **(B)**, and waveform **(C)** experiments. Here *Q* is equivalent to *Q*_0_. For amplitude, the results show a linear increase with increasing intensity. Frequency experiments show a window (50–180 Hz) that seems to produce similar outcomes and differs to the other frequencies (15, 250, and 350 Hz). Waveform ED results show more similarity between *Rect* 1.5 × *Q* and other waveforms at 2 × *Q*.

In each experimental paradigm, we tried to alter only one parameter in order to better control the test conditions. Initially, we focused on adjusting the stimulation amplitude. Despite the key role that DBS amplitude plays in DBS calibration procedures, multiple surveys have shown that only fine-tuning the amplitude can lead to side effects such as gait problems, movement interruption and speech impairments (Törnqvist et al., 2005; Moreau et al., 2008; Tommasi et al., 2008; Xie et al., 2012; Tripoliti et al., 2014; Dayal et al., 2017). It has been reported that the total electrical energy delivered (TEED) has a significant impact on STN-DBS treatments (Schor and Nelson, 2019). TEED can be calculated as follows:

$$T\,E\,E\,D=\frac{I^{2}\times f\times P\,W}{Z}\,\left(1\right)$$

where *I* is the current amplitude, *f* stimulation frequency, *PW* pulse-width and *Z* is the electrode-tissue impedance. A study by Schor and Nelson (2019) has reported that the current amplitude has a linear effect on hemi-PD mice’s movement velocities. Our finding also corroborates a strong linear relationship between the amplitude and all the three features we defined (rotation, area under the curve, and total distance). Despite the presented differences between waveforms, this linear relationship remains valid for every waveform we tested. In order to quantify the incrementing similarity across all amplitudes, we computed the Euclidean distance (ED) between the total rotation of all amplitude pairs (see Figure 7A and Table 5).

**TABLE 5:** Euclidean distance of rotation trajectories between amplitude pairs under *Rect* stimulation.

	***Q*_0_**	**1.25 × *Q*_0_**	**1.5 × *Q*_0_**	**1.75 × *Q*_0_**	**2 × *Q*_0_**
*Q*_0_	0				
1.25 × *Q*_0_	5.48	0			
1.5 × *Q*_0_	7.38	2.66	0		
1.75 × *Q*_0_	14.85	9.46	8.10	0	
2 × *Q*_0_	23.6	18.20	16.59	9.25	0

Variations in frequency smaller than 50 Hz are repeatedly reported to worsen patients’ symptoms (Moro et al., 2002; Timmermann et al., 2004; Eusebio et al., 2008; Dayal et al., 2017). Frequencies from 50 to 130 Hz are known to cause significant improvements in the symptoms with the best outcomes achieved within the 130–180 Hz range (Moro et al., 2002). It has been shown that the effectiveness of DBS decreases as the frequency surpasses 185 Hz. Clinical studies on stimulation with frequencies above 185 Hz have reported side effects such as lower limb dyskinesias and the sensation of having a heavy head (Moro et al., 2002; Dayal et al., 2017). Studies comparing 60 and 130 Hz stimulations have demonstrated that by maintaining the same total delivered energy, 60 Hz stimulation results in improvements in the occurrence of freezing gait, axial symptoms, akinesia, bradykinesia, and dyskinesias (Moreau et al., 2008; Xie et al., 2012; Khoo et al., 2014; Ramdhani et al., 2015). However, other studies exist which have been unable to show significant differences between 130 Hz and 60–80 Hz stimulation (Brozova et al., 2009; Ricchi et al., 2012; Sidiropoulos et al., 2013; Phibbs et al., 2014). Our results demonstrate similar behavioral effects with a DBS frequency between 50 and 180 Hz. Thus the stimulation’s therapeutic frequency window appears to be valid in the 6-OHDA rat model as well. However, rotational behavior was still comparable with a frequency between 250 and 350 Hz, suggesting a ceiling or saturation effect. Our similarity assessments between the frequency pairs show the clear correspondence within the therapeutic frequency windows (see Figure 7 and Table 6).

**TABLE 6:** Euclidean distance (ED) of rotation trajectories between frequency pairs.

	***Q*_0_ (15 Hz)**	**3.3 × *Q*_0_ (50 Hz)**	**6.6 × *Q*_0_ (100 Hz)**	**8.6 × *Q*_0_ (130 Hz)**	**12 × *Q*_0_ (180 Hz)**	**16.6 × *Q*_0_ (250 Hz)**	**23.3 × *Q*_0_ (350 Hz)**
*Q*_0_ (15 Hz)	0						
3.3 × *Q*_0_ (50 Hz)	19.04	0					
6.6 × *Q*_0_ (100 Hz)	16.74	4.08	0				
8.6 × *Q*_0_ (130 Hz)	17.94	2.93	2.93	0			
12 × *Q*_0_ (180 Hz)	17.47	2.32	2.66	2.50	0		
16.6 × *Q*_0_ (250 Hz)	29.63	11.43	13.09	12.53	12.72	0	
23.3 × *Q*_0_ (350 Hz)	30.50	11.85	14.10	13.24	13.40	2.60	0

Reducing the charge per phase in DBS without losing the treatment quality is a desirable goal. Lowering the injected charge would prolong the implantable pulse generator’s (IPG) battery life and decrease the probability of tissue damage. Therefore, taking a closer look at the pulse-shape and waveform of the stimulation promises worthwhile for any DBS application. Several studies have attempted to stimulate neural tissue using non-*Rect* waveforms. Among others, *Tri*, *Sine*, gaussian shapes and a few more have been tested and it has been suggested that there might be a more energy efficient waveform than the standard biphasic *Rect* pulses (Sahin and Tie, 2007; Foutz and McIntyre, 2010; Jezernik et al., 2010; Wongsarnpigoon and Grill, 2010).

The computer simulation study done by Sahin and Tie (2007) showed that linearly decreasing ramps (Lin.Dec.) were more efficient than *Rect* pulse shapes. Another study done by Wongsarnpigoon and Grill (2020), involving a computational model of mammalian axons and tested *in vivo* in cat sciatic nerve, showed that a genetic algorithm (GA) optimized waveform could outperform the conventional waveform shapes used for neural tissues. And once again, among the other waveforms, decreasing ramp and decaying exponentials were the most similar waveforms to the GA waveform (difference = ∼20%) when PW_*anodic*_ and PW_*cathodic*_ were similar and the PW = 0.05 ms (Wongsarnpigoon and Grill, 2010).

In our study, normalizing the injected charges to an individualized *Q*_0_, thus taking biological variations into account, demonstrated only non-significant differences in outcome of alternative waveforms compared to the standard *Rect.* However, when we look at the 1.5 × *Q*_0_ and 2 × *Q*_0_ charge levels, we see that *Rect* was able to induce more than twice the number of rotations than the other waveforms. In fact, the rotations caused by the 1.5 × *Q*_0_ *Rect* waveform were comparable to the rotation results of the other waveforms with 2 × *Q*_0_ charge. To quantify and assess this similarity the ED was measured between waveform-amplitude pairs (see Figure 7C and Table 7). The *Tri* waveform with 2 × *Q*_0_, showed the least ED (highest similarity) to the *Rect* 1.5 × *Q*_0_ (as highlighted in yellow in Table 7).

**TABLE 7:** Euclidean distance of rotation trajectories between waveform pairs.

	***Rect* Q**	***Rect* 1.5 × *Q***	***Rect* 2 × *Q***	***Sine Q***	***Tri Q***	***Lin.Dec*. *Q***	***Sine* 1.5 × *Q***	***Tri* 1.5 × *Q***	***Lin. Dec*. 1.5 × *Q***	***Sine* 2 × *Q***	***Tri* 2 × *Q***	***Lin. Dec*. 2 × *Q***
*Rect Q*	0	4,216	6,879	252	298	306	1,305	1,666	254	3,435	4,461	3,837
*Rect* 1.5 × *Q*	4,216	0	2,715	4,194	3,990	4,339	2,954	2,575	4,338	938	488	577
*Rect* 2 × *Q*	6,879	2,715	0	6,859	6,658	7,005	5,630	5,247	7,007	3,535	2,479	3,102

## Conclusion

Neither PW, nor polarity, wave asymmetry or interphase intervals were varied in this study (Stieger et al., 2020). We thus cannot claim our systematic investigation of different STN-DBS parameters in the hemi-parkinsonian rat to be complete, yet we were already able to show that the transiently induced rotation behavior provides a valuable readout to assess DBS parameters in the hemi-PD rat. Since each single parameter may influence the behavioral result, we suggest to consider all of them with an optimization procedure. During our waveform variation experiments, charges were kept consistent by adjusting the amplitude of the pulses and keeping the PW intact. One can keep the injected charge comparable among the waveforms by using the same amplitude and adjusting the PW.

For future testing, experimental paradigms that randomize the order of each parameter sequence should be considered to avoid possible bias toward stimulation sequences and behavioral outcome. All in all, we suggest the use of the versatile hemi-PD rat model or other similar models to finally sample the full size of DBS’s electrical parameter space for the benefit of the patients.

## Data Availability Statement

The raw data supporting the conclusions of this article will be made available by the authors, without undue reservation, to any qualified researcher.

## Ethics Statement

The animal study was reviewed and approved by Animal Care Committee of the University of Freiburg under approval G15/031.

## Author Contributions

SM designed and conducted experimental and analyzed the results. UH supervised the experimental and analysis work. SM, OB, and UH wrote and reviewed the manuscript.

## Conflict of Interest

The authors declare that the research was conducted in the absence of any commercial or financial relationships that could be construed as a potential conflict of interest.
